# A 2013 workshop: vaccine and drug ontology studies (VDOS 2013)

**DOI:** 10.1186/2041-1480-5-16

**Published:** 2014-03-20

**Authors:** Cui Tao, Yongqun He, Sivaram Arabandi

**Affiliations:** 1 School of Biomedical Informatics, The University of Texas Health Science Center, Houston, TX, USA; 2 Unit for Laboratory Animal Medicine, Department of Microbiology and Immunology, and Center for Computational Medicine and Bioinformatics, University of Michigan Medical School, Ann Arbor, MI, USA; 3 Ontopro LLC, Houston, TX, USA

## Abstract

The 2013 “Vaccine and Drug Ontology Studies” (VDOS 2013) international workshop series focuses on vaccine- and drug-related ontology modeling and applications. Drugs and vaccines have contributed to dramatic improvements in public health worldwide. Over the last decade, tremendous efforts have been made in the biomedical ontology community to ontologically represent various areas associated with vaccines and drugs – extending existing clinical terminology systems such as SNOMED, RxNorm, NDF-RT, and MedDRA, as well as developing new models such as Vaccine Ontology. The VDOS workshop series provides a platform for discussing innovative solutions as well as the challenges in the development and applications of biomedical ontologies for representing and analyzing drugs and vaccines, their administration, host immune responses, adverse events, and other related topics. The six full-length papers included in this thematic issue focuses on three main areas: (i) ontology development and representation, (ii) ontology mapping, maintaining and auditing, and (iii) ontology applications.

## Introduction and background

Drugs and vaccines have been critical to prevent and treat human and animal diseases. Work in both (drugs and vaccines) areas is closely related - from preclinical research and development to manufacturing, clinical trials, government approval and regulation, and post-licensure usage surveillance and monitoring. Many drug and vaccine related ontologies have already been or are being developed for different use cases and applications. The 2013 “Vaccine and Drug Ontology Studies” workshop (VDOS 2013) workshop series aims to become an international forum for researchers to identify, propose, and discuss solutions for important research problems in ontology representation and analysis of vaccine and drug formation and preparation, administration, function mechanisms, and induced host immune responses. The immune responses can be positive responses for prevention and/or treatment of a disease, or can be negative responses, i.e., adverse events. This workshop aimed to support the deeper understanding of vaccine and drug mechanisms and effects.

VDOS 2013 was held on July 7, 2013, at Montreal, Qc, Canada. This workshop was part of the fourth International Conference on Biomedical Ontology (ICBO 2013). The workshop attracted interest from many international attendees, including paper presenters, senior academic and government scientists, postdoctoral fellows, and graduate students. After a rigorous peer review process (all submissions have been reviewed by at least three independent reviewers), six full-length papers and three short-length papers were accepted for proceeding paper publications and oral presentations in the workshop. After one additional round of independent peer reviewing by the workshop co-organizers and the journal editors, the selected six full-length papers were extended and accepted for publication in the current issue of the Journal of Biomedical Semantics (JBMS).

The VDOS-2013 workshop is the 2nd in this series. The first workshop of the series was organized as the “Vaccine and Drug Ontology in the Study of Mechanism and Effect” workshop (VDOSME 2012) [1] on July 21, 2012, at Graz, Germany, as part of the third International Conference on Biomedical Ontology (ICBO 2012). For this year, the name has been changed to “Vaccine and Drug Ontology Studies (VDOS)” to reflect the expansion in the scope to more than just mechanism and effect. The workshop series also covers vaccine and drug-related clinical data representation and analysis, including clinically reported vaccine and drug adverse events.

## Summary of selected papers in the thematic issue

The six papers selected for this thematic issue are extended versions of the original full-length papers presented at the VDOS 2013 [2-7]. These papers cover a wide range of topics including ontology development and representation, ontology mapping, maintaining and auditing, and ontology applications.

In the area of ontology development and representation, Lin and He introduced their Ontology of Genetic Susceptibility Factors (OGSF) for representing the susceptibility factors for post vaccination events using a formal ontological mechanism [2]. OGSF is aligned with the Basic Formal Ontology (BFO). This paper ontologically defines two core OGSF terms ‘genetic susceptibility’ and ‘genetic susceptibility factor’ and the design pattern for representing genetic susceptibility to a vaccine adverse event. Two use cases related to the genetic susceptibility to adverse events following vaccination of an influenza vaccine or smallpox vaccine were studied using the OGSF ontology. Hanna et al built the Drug Ontology (DrOn) to model drug information for comparative-effectiveness research [3]. DrOn is represented in OWL2 and covers drug information derived from three different sources (RxNorm, ChEBI, and PRO). Although DrOn was originally designed for comparative-effectiveness research studies, it can also service many other use cases in the biomedical domain. Marcos and He [4] developed the Ontology of Vaccine Adverse Events (OVAE). OVAE was built as an extension of the Vaccine Ontology (VO) and the Ontology of Adverse Events (OAE). It represents and classifies the adverse events recorded in package insert documents of commercial vaccines licensed by the USA Food and Drug Administration (FDA). OVAE will be very useful in supporting rational VAE prevention and treatment and benefits public health.

In the area of ontology mapping, Winnenburg, et al. tried to map the terms in (Anatomical Therapeutic Chemical (ATC) and Medical Subject Headings (MeSH) through the Unified Medical Language System (UMLS) [5]. Both lexical-based and instance-based mapping were performed, which yielded hundreds of new mappings between the two terminology systems. The alignment between ATC and MeSH can be critical for drug evaluation and safety studies as well as for pharmacogenetic research. On one hand, MEDLINE literature is indexed using MeSH. On the other hand, adverse drug events are usually analyzed in reference to ATC. In order to integrate drug information from these different sources, a reliable alignment between ATC and MeSH is very much desired.

In the area of ontology applications, Doulaverakis et al. present a semantic framework to discover drug-drug and drug-disease interactions [6]. They use SKOS to represent the semantics of the medical classification for drug relevant information derived from ICD-10, Unique Ingredient Identifier (UNII), ATC, and the International Virus Taxonomy (IVT). Rule-based reasoning approaches were then used to identify drug recommendations. Zhang et al. introduce a novel approach that combines ontologies and network analysis technologies for identifying new associations among vaccines, genes, and diseases [7]. The authors leverage data extracted from MEDLINE and represented this information using Resource Description Framework (RDF). This RDF graph can then be viewed as a network to perform network analysis.

## Workshop presentations and discussions

In the workshop, the six full-length papers described above were orally presented. In addition, three short papers were accepted for short oral presentations. Zhu et al. introduces their work on building a drug and drug class network derived from multiple drug terminological resources, such as ATC, National Drug File Reference Terminology (NDF-RT), RxNorm, and Structured Product Label (SPL) [8]. He et al. investigated how to audit the redundancies caused by importing top-level ontologies [9]. More specifically, they studied the redundancies in Drug Discovery Investigations ontology (DDI) when importing BFO. Hall et al. introduces their software that supports extracting new drug information from drug structured product labels (SPL) to update the DrOn [10].

During the discussion session, we discussed two main areas – (a) mapping between different drug models (6 papers), and (b) adverse events detection and analysis (3 papers). The major focuses on drug ontologies themselves (including models that contribute greatly in this area), their goals, and the challenges in aligning them, show that some of the preparatory work still needs to be done to continue the research into adverse events. A number of different approaches to drug mapping were discussed – lexical, ingredient based, using chemical structures and by exploiting networks (e.g. via the UMLS). While each provided their unique benefits, there was concurrence on the need for using more than one method to improve the quality of mapping. Furthermore, these papers also demonstrated the need for enhancing the definitions (logical and textual) of the terms and the relations both for improved human understanding as well as for better integration between them. The two papers focusing on adverse event detection and analysis also highlighted some of the gaps and shed light on areas for further ontology development efforts. These studies also demonstrated promising usages and advantages of ontology in standardizing, integrating, and analyzing adverse event data.

Overall, the VDOS 2013 workshop provided an ideal platform for ontology researchers and users to present and discuss the progresses and issues in the development and applications of ontologies related to vaccines and drugs. Positive feedbacks were obtained.

## Competing interests

The authors declare that they have no competing interests.
